# Can protection motivation theory predict protective behavior against ticks?

**DOI:** 10.1186/s12889-023-16125-5

**Published:** 2023-06-22

**Authors:** Mette Frimodt Hansen, Pelle Korsbaek Sørensen, Anja Elaine Sørensen, Karen Angeliki Krogfelt

**Affiliations:** 1 Department of Science and Environment, Roskilde University, Universitetsvej 1, Roskilde, DK-4100 Denmark; 2 Research Centre for Health Promotion, Roskilde University, Roskilde, Denmark; 3 Research and Development, Centre for Nursing, University College Absalon, Roskilde, Denmark

**Keywords:** Protection motivation theory, Ixodes ricinus, Lyme borreliosis, Tick-borne encephalitis, Risk perception, Protective behavior

## Abstract

**Background:**

Cases of reported tick-borne diseases in humans have increased over the past decades. Strategies informing the public about ticks, their associated diseases, and preventive measures are often highlighted as important in limiting pathogen transfer and disease. However, knowledge about the motivation for people to apply preventative measures is sparse.

**Methods:**

The aim was to examine if Protection Motivation Theory, a model of disease prevention and health promotion, can predict the use of protective measures against ticks. Ordinal logistic regression and Chi-square tests were used on data from a cross-sectional survey with respondents from Denmark, Norway, and Sweden (n = 2658). We examined the effect of (1) the perceived seriousness of tick bites, Lyme borreliosis (LB), and tick-borne encephalitis (TBE), and (2) the perceived probability of getting a tick bite, Lyme borreliosis, and tick-borne encephalitis on protection against ticks. Finally, we examined if there was an association between the use of a protective measure and the perceived efficacy of that measure.

**Results:**

The perceived seriousness of a tick bite and LB significantly predict who is more likely to apply protective measures for all three countries combined. The perceived seriousness of TBE did not significantly predict the level of adoption of protective measures applied by respondents. The perceived likelihood of getting a tick bite within the next 12 months and the perceived likelihood of getting LB if bitten by a tick significantly predicted the application of protective measures. However, the increases in the likelihood of protection were very small. The application of a certain type of protection was always correlated with the perceived efficacy of the same protective measure.

**Conclusion:**

Some variables of PMT may be used to predict the level of adoption of protection applied against ticks and tick-borne diseases. We found that the perceived seriousness of a tick bite and LB significantly predict the level of adoption protection. The perceived likelihood of getting a tick bite or LB also significantly predicted the level of adoption of protection, although the change was very small. The results regarding TBE were less clear. Lastly, there was an association between applying a protective measure and the perceived efficacy of the same measure.

**Supplementary Information:**

The online version contains supplementary material available at 10.1186/s12889-023-16125-5.

## Background

Vector-borne diseases are caused by pathogenic microorganisms transmitted by a vector such as arthropods and account for more than 17% of all infectious diseases worldwide [1]. Ticks are important vectors of pathogen transmission in Europe and the U.S [2], [3]. Examples of human tick-borne diseases (TBDs) are the bacterial disease Lyme borreliosis (LB), and the viral disease Tick-borne encephalitis (TBE).

An unweighted mean incidence rate for LB in Western Europe based on reported incidences in the literature has been estimated to be 56.3 per 100,000 persons equating to approximately 232,125 cases per year [4]. In the U.S., estimations based on insurance claims suggest an incidence rate of 49–88 per 100,000 [5] or an annual number of 476,000 Americans being diagnosed and treated for LB [5]. These numbers indicate a potentially large burden on the health care system and stress the potential risk of contracting LB or other TBDs in areas with ticks in Europe and the U.S. Misdiagnosis of Lyme neuroborreliosis (LNB) in primary care [6], treatment delay [7] and the possible negative outcome of treatment delay [8], [9] have been reported. A cost of 5500€ including health care costs and social benefit costs per LNB patient has been estimated in Sweden [10].

The risk of contracting a TBD may be minimized by several human actions [11], and the prevention of tick bites and minimization of feeding time of the tick is crucial in limiting pathogen transfer [12], [13]. Several protective measures, such as tucking trousers into socks, using tick repellents, and checking the body for ticks, are often recommended to protect oneself against ticks and TBDs, although the evidence of actual effectiveness of these measures is difficult to assess [14].

Several studies have examined the knowledge of ticks, TBDs, and protective measures applied against ticks by people in several countries, all pointing to the conclusion that knowledge about ticks, TBDs, and protective measures is low and level of adoption of protection could be improved [15–19]. All these studies emphasize the need for education and awareness among the public, which is suggested to lead to behavioral changes favouring better protection against TBDs.

However, although necessary for the understanding of a potential risk, education and awareness are not the only variables needed to enforce behavioral changes [20]. Another variable that has long been regarded as an adaptive response protecting one against danger, both in psychology and biology, is fear [20], [21]. Protection Motivation Theory (PMT**)**, a model of disease prevention and health promotion, is partly built on the concept of fear appeal [20], and is defined as a message based on persuading the receiver into a behavioral change based on the arousal of fear [22]. Based on Rogers’ later revisions [23], PMT has been extended and there are additions to the focus of fear appeal, and PMT has also been used to evaluate persuasive communications and to predict health behaviour [24]. However, the data set used in this article is coherent with the original model.

The variables of the original PMT are magnitude of noxiousness, probability of occurrence, and efficacy of a recommended response [20]. Thus, in health decision making, the motivation for a possibly inconvenient, behavioral change is dependent on the perceived level of noxiousness, the perceived likelihood of a noxious event occurring, and a trust that a recommended response (e.g. protection against ticks) will actually be beneficial [25]. If this is not achieved, no protection will be encouraged and hence, no change in behavior will occur [20].

Indications of PMT being applicable to different fields, including areas beyond health-related issues, have been reported [26]. PMT has also been applied to predict protective behaviour regarding several vector-borne diseases among respondents in Africa, Asia, and the U.S [27–29]. A meta-analysis including 65 studies [30] and a quantitative review [31] on PMT suggest that components of the model may be relevant in individual and community health-related interventions.

Previous studies from Scandinavian countries on protection against TBDs have mostly focused on the knowledge and risk perception of ticks and their associated diseases, as well as how well people protect themselves separately [15], [32]. What is important in public health strategies against ticks, however, is an understanding of what factors motivate people to adopt protective behavior against ticks. Studies from the U.S., U.K., and Sweden have found an association between perceived severity of LB, perceived likelihood of contracting a TBD, and tick bites and performing a tick check [33–35].

Since PMT can be used as a model to predict health behavior [36], the aim of this study was to examine if variables of the original PMT from 1975 can predict who protects themselves against tick bites. The variables considered were the perceived seriousness and likelihood of a tick bite, LB, and TBE in Denmark, Norway, and Sweden. Further, we examined if there is an association between a protective measure reported to be used by a respondent and the perceived efficacy of the same protective measure.

We hypothesized that a higher perceived seriousness and likelihood of a tick bite, LB, or TBE to occur will lead to an increase in level of adoption of protection. Further, we hypothesized that the use of a protective measure is associated with perceived efficacy of the same measure.

Since personal protective behavior is often highlighted as a crucial strategy in reducing the TBD incidence rate, [1], [33], [37–39], determining which factors predict how well and often a person protects themself is relevant in the development of optimal prevention strategies of LB, TBE, and other TBDs.

## Methods

### Survey and study design

A cross-sectional survey was developed and conducted in 2016 in Denmark, Norway, and Sweden as part of a joint Scandinavian research project (ScandTick Innovation). Thorough descriptions of the study design, study sample, the survey, data collection, and rate of participation have been provided previously [15], [32], [40]. Although some of the questions have been examined and reported previously, they have been analyzed in a different context and the analyses in this study are new. The same data was used but new analyses were run in order to examine the application of PMT. In this study, we aim at testing PMT to examine if the theory can be applied to tick protection behavior and hence gain a better understanding of possible variables that may affect protection motivation. To our knowledge, this was not examined in the previous publications.

We examine the effect of one or multiple independent variables on the dependent variable “protection” which is a three-point ordinal scale that measures levels of adoption of protection based on six types of protective measures (Table 1).

**Table 1:** Types of protective measures examined

Protective measures examined
1. Wearing clothes that cover legs and arms
2. Using mosquito and tick repellents
3. Tucking trousers in to socks
4. Avoiding walking in tall grass and near bushes
5. Checking body and clothes while visiting areas with ticks
6. Checking body and clothes after visiting areas with ticks

The three levels of adoption of protection are: rarely/never use any, often/always use 1–2, and often/always use 3 or more, as previously defined [15]. We focus on a subset of questions from the survey, that we defined as matches to the variables of PMT. That is (1) questions regarding protection which corresponds to the behavioral response we seek to examine, i.e. the dependent variable “protection” (q20 ) and the perceived efficacy of a protective response (q22); and (2) the independent variables “perceived seriousness a tick bite”, “LB”, or “TBE” (q23A-C) which corresponds to the magnitude of noxiousness of an event; and (3) the perceived probability of getting a tick bite (q24), getting LB if bitten (q25), and getting TBE if bitten (q26) which corresponds to the probability of a noxious event occurring. Questions analysed in this study can be found in Appendix 1 in supplementary information.

The variables of perceived seriousness, i.e., “Perceived seriousness of a tick bite” (n = 2658), “Perceived seriousness of LB” (n = 2562), and “Perceived seriousness of TBE” (n = 1850) were assessed by a question where the respondent had answered on a 0–10 scale how serious they believed it was to get a tick bite. ‘Don’t know’ was also an option but was excluded from analyses since we were examining the effect of different perceptions on applying a protective measure. ‘Don’t know’ was answered by 1.4% for tick bite, 1.3% for LB, and 3% for TBE. The variables were binned during analysis to a three-point scale with 0–3 = not serious, 4–6 = serious, and 7–10 = very serious due to some values having too few responses. Perceived probability of getting a tick bite (n = 2650), LB (n = 2519), or TBE (n = 1815) was reported on a scale from 0 to 100 with 0 being “I’m absolutely certain I will not get bitten” and 100 being “I’m absolutely certain I will get bitten”.

Additional confounding variables included in the analyses were gender (n_female_ = 1403, n_male_ = 1255, people responding ‘other’ or ‘I choose not to respond’ (n = 10) were excluded from the analyses) and country (Denmark (n = 781), Norway (n = 786), and Sweden (n = 1091). Previous analyses of age groups have shown statistically significant differences between some age groups in “likelihood of contracting LB and TBE” and“perceived seriousness of tick bite, LB, and TBE” and protection [15], [32]. The use of different age groups in these articles indicates that how the age group is defined may impact the results. We performed a cumulative odds orginal logistic regression with age group (18–29, 30–44, 45–59, 60+) [32] as an independent variable and level of adoption of protective measures as the dependent variable (p = .326) and the variable was not included in further analyses.

### Statistics

Cumulative odds ordinal logistic regression with proportional odds was used to evaluate if the independent variables (perceived seriousness of a tick bite / LB / TBE and perceived likelihood of getting a tick bite / LB / TBE) as well as the interactions of these with gender and country have a statistically significant effect on the dependent variable ‘protection’. Chi-square tests of independence were performed to assess the association between the categorical variables “reported use of a protective measure” (q20) and “the believed efficacy of the same protective measure” (q22) for all three countries combined and separately. The correlation between the categorical variables were checked using Cramer’s V for the countries combined. The statistical analyses were performed using IBM SPSS statistics version 27 and GraphPad Prism 9. A p-value < 0.05 was considered statistically significant.

## Results

### The effect of perceived seriousness of a tick bite and LB on the use of protective measures

The perceived seriousness of a tick bite (Wald^2^(2, n = 2658) = 64.109, p = < 0.001) and LB (Wald^2^(2, n = 2562) = 23.486, p = < 0.001) significantly predicted the level of adoption of protection.

**Fig. 1:**
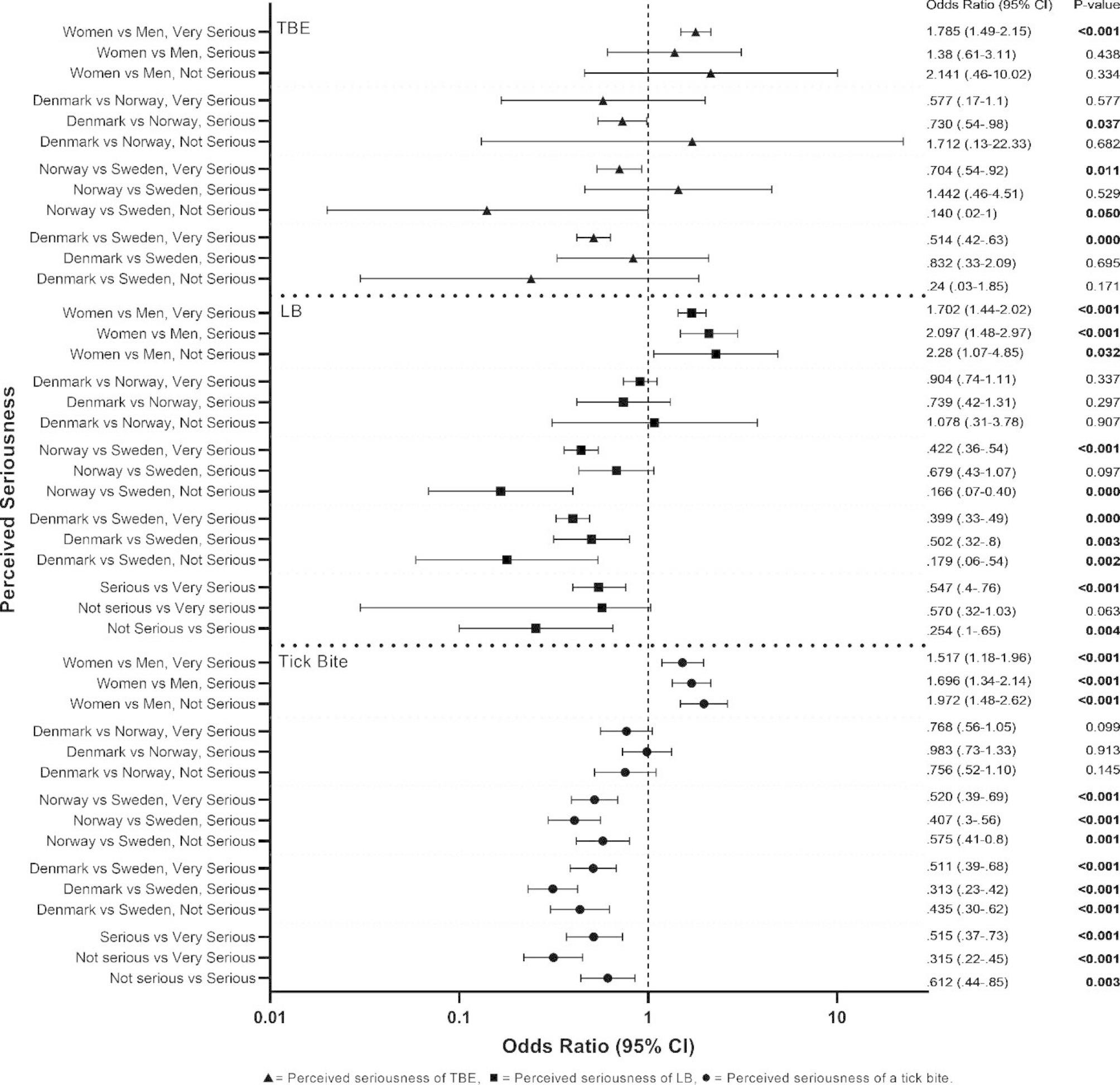
The odds ratios of the effect of perceived seriousness of a tick bite, LB, or TBE on the level of adoption of protection applied by respondents. The effect of the interaction of country and perceived seriousness and interaction of gender and perceived seriousness on level of adoption of protection are included. P-values < 0.05 are considered significant and are highlighted in bold. The reference variable is always the last, e.g., ‘Women vs Men’ ‘Very serious’: The odds of women protecting themselves ‘often/always’ are 1.785 times that of men, when both perceive TBE to be ´Very serious’.

Respondents who answered that tick bites and LB are not serious are less likely to protect themselves compared to those who think they are very serious. Women were more likely than men to use protective measures regardless of how serious they thought tick bites or LB were. People from Denmark and Norway were less likely to protect themselves compared to people from Sweden, regardless of how serious they thought the diseases were (Fig. 1; Tables 2 and 3).

**Table 2:** The effect of perceived seriousness of a tick bite on applying protective measures against ticks

Country	Perceived seriousness of tick bite	Wald^2^(df)	Odds Ratio (95% CI)		P value
Denmark	Not serious	20.653(1)		0.435 (0.304-0.623)	< 0.001
Denmark	Serious	58.218(1)		0.313 (0.232-0.422)	< 0.001
Denmark	Very serious	21.848(1)		0.511 (0.386-0.677)	< 0.001
Norway	Not serious	11.216(1)		0.575 (0.416-0.795)	0.001
Norway	Serious	30.653(1)		0.407 (0.296-0.560)	< 0.001
Norway	Very serious	20.956(1)		0.520 (0.393-0.688)	< 0.001

*Sweden is the reference country

**Table 3:** The effect of perceived seriousness of LB on applying protective measures against ticks

Country	Perceived seriousness of LB		Wald^2^(df)	Odds Ratio (95% CI)	P value
Denmark		Not serious	9.247(1)	0.179 (0.059-0.542)	0.002
Denmark		Serious	8.595(1)	0.502 (0.316-0.796)	0.003
Denmark		Very serious	77.813(1)	0.399 (0.325-0.490)	< 0.001
Norway		Not serious	15.972(1)	0.166 (0.069-0.400)	< 0.001
Norway		Serious	2.757(1)	0.679 (0.430-1.072)	0.097
Norway		Very serious	59.492(1)	0.442 (0.359-0.544)	< 0.001

*Sweden is the reference country

### The effect of perceived seriousness of TBE on the use of protective measures

The perceived seriousness of TBE alone did not significantly predict level of adoption of protection, Wald^2^(2) = 3.429, p = .180.

Respondents from Denmark who believe TBE is very serious are less likely to protect themselves compared to people from Sweden with similar perceived seriousness. Similarly, people from Norway who think TBE is not serious are less likely to protect themselves compared to people from Sweden who also think it’s not serious. When it comes to gender, the only significant difference was when both men and women thought TBE was very serious, where women were more likely to protect themselves compared to men (Fig. 1).

### The effect of perceived likelihood of getting a tick bite within the next 12 months on the use of protective measures

A one-unit increase in perceived likelihood of getting a tick bite within the next 12 months (expressed in percentage) was associated with an increase in the odds of using more protective measures, with an odds ratio of 1.009 (95% CI 1.006–1.013).

**Fig. 2:**
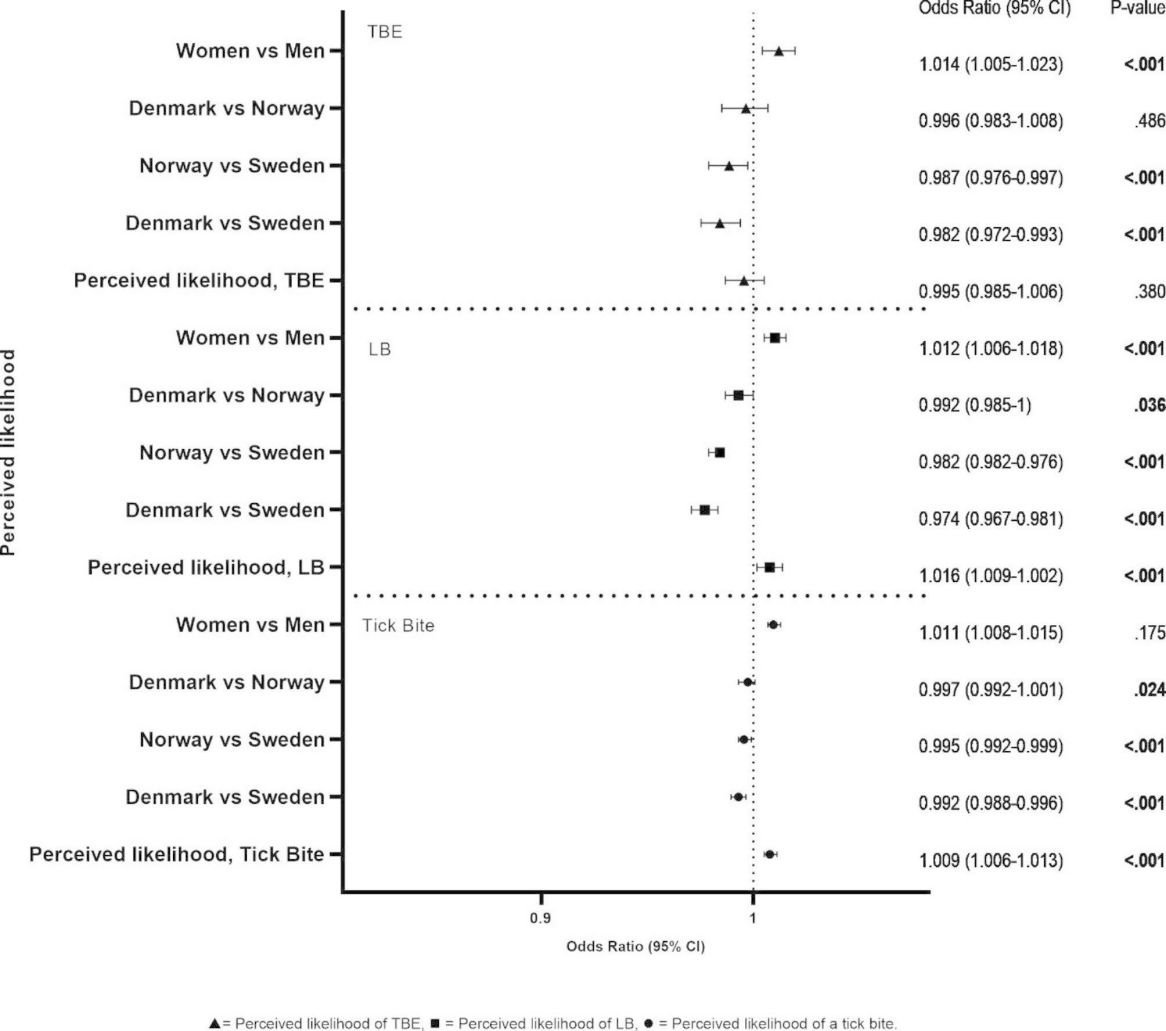
The odds ratios of the effect of perceived likelihood of getting a tick bite within the next 12 months, getting LB if bitten by a tick, or getting TBE if bitten by a tick on the level of adoption of protection applied by respondents. P-values < 0.05 are considered significant and are highlighted in bold. The reference variable is always the last, e.g., if: ‘Women vs Men’: The odds of women protecting themselves ‘often/always with more than three types’ are 1.014 times that of men, when perceived likelihood of TBE is equal.

Danes and Norwegians are less likely to protect themselves more for every one-unit increase in the perceived risk of getting a tick bite compared to Swedes (Fig. 2).

Women are more likely to protect themselves than men when the perceived risk of getting a tick bite within the next 12 month increases with an odds ratio of 1.011 (95% CI 1.008–1.015, p < .001) for every one unit increase.

### The effect of perceived likelihood of getting LB if bitten by a tick on the use of protective measures

A one-unit increase in likelihood of getting LB if bitten by a tick was associated with an increase in the odds of applying more protective measures, with an odds ratio of 1.016 (95% CI 1.009–1.022) Wald^2^(1) = 21.961, p < .001.

Danes and Norwegians are less likely to protect themselves when the perceived likelihood of getting LB if bitten by a tick increases by one unit compared to Swedes.

Danes are slightly less likely to protect themselves compared to Norwegians when the perceived likelihood of getting LB if bitten by a tick increases by one unit.

Women are 1.2% more likely to protect themselves more than men when the perceived likelihood of getting LB if bitten by a tick increases by one unit (Fig. 2).

### The effect of perceived likelihood of getting TBE if bitten by a tick on the use of protective measures

A one-unit increase in ‘perceived likelihood of TBE if bitten by a tick’ alone was not associated with an increase in the odds of applying more protective measures, with an odds ratio of 0.995 (95% CI 0.985–1.006).

Danes and Norwegians are less likely to protect themselves more when the perceived likelihood of getting TBE if bitten by a tick increases by one unit compared to Swedes, while Danes and Norwegians are equally likely. (Fig. 2).

Women are 1.4% more likely to protect themselves than men when the perceived likelihood of getting TBE if bitten by a tick increases by one unit (Fig. 2).

### Association between use of protective measures and perceived efficacy of the same type of protective measure

#### Denmark, Norway, and Sweden combined

**Fig. 3:**
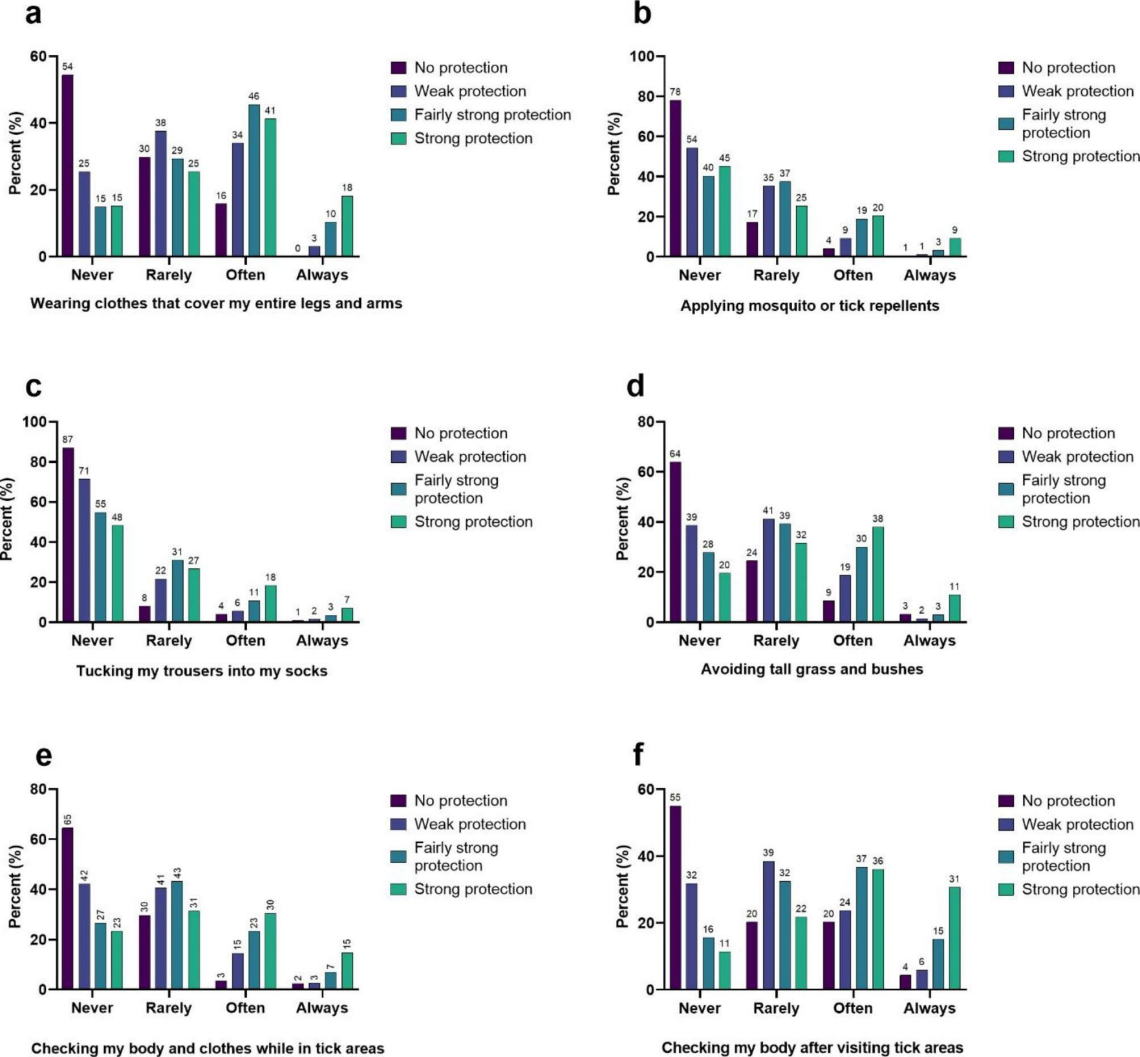
Percentage distribution of the perceived efficacy (no protection, weak protection, fairly strong protection, or strong protection) of six types of protective measures (a-f) and how often a respondent applies that same protection when in areas with ticks (never, rarely, often, or always. The values are from respondents from Denmark, Norway, and Sweden combined.

There was a significant association between the categories “Never, rarely, often, and always” in response to each protective measure and “No protection, weak protection, fairly strong protection, and very strong protection” regarding the same protective measure (Fig. 3; Table 4).

When the countries were examined separately, all variables were significantly associated (Table 4).

**Table 4:** Chi-square tests examining the association between applying a protective measure and perceived efficacy of the same measure and the strength of the association (Cramer’s v)

	Denmark (DK)		Norway (NO)		Sweden (SE)		DK, NO, and SE combined	
	P value	Cramer’s v	P value	Cramer’s v	P value	Cramer’s v	P value	Cramer’s v
Wearing clothes that cover legs and arms	< 0.001	0.157	< 0.001	0.130	< 0.001	0.144	< 0.001	0.137
Use mosquito repellents	< 0.001*	0.130	< 0.001	0.197	< 0.001	0.182	< 0.001	0.170
Tuck trousers in to socks	< 0.001	0.134	< 0.001	0.130	< 0.001	0.132	< 0.001	0.123
Avoid tall grass and walking near bushes	< 0.001	0.179	< 0.001	0.174	< 0.001	0.157	< 0.001	0.162
Check body and clothes while visiting areas with ticks	< 0.001	0.177	< 0.001	0.177	< 0.001	0.194	< 0.001	0.179
Check body and clothes after visiting areas with ticks	< 0.001	0.190	< 0.001	0.213	< 0.001	0.160	< 0.001	0.176

*25% expected counts less than 5

Notes: Perceived efficacy (no protection, weak protection, fairly strong protection, or strong protection) and how often a respondent apply that same protection when in areas with ticks (never, rarely, often, or always)

## Discussion

We examined if PMT is applicable in predicting adoption of protective behavior against ticks in three Scandinavian countries. The independent variables ‘perceived seriousness of a tick bite or LB’ significantly predicted who was more likely to apply protective measures except when LB was considered ‘not serious’ versus ‘serious’ for all three countries combined. Hence, a person who believes a tick bite is ‘serious’ or ‘very serious’ is more likely to apply more protective measures than a person who believes a tick bite is ‘not serious’. The perceived seriousness of TBE did not significantly predict level of adoption of protective measures applied by respondents.

Motivation to protect one-self is dependent on several variables [20]. Here we observe a difference between a well-known (LB) and less well-known (TBE) disease, as overall, more respondents had heard of LB than of TBE [32]. In Denmark, Norway, and Sweden, TBE virus is limited to certain endemic areas [41–44], whereas *Borrelia burgdorferi* s.l. is more widespread [45] which could explain why, even though TBE may be perceived as being serious, it will not lead to adoption of protection since only a small part of the respondents may be visiting areas that are TBEV risk areas.

Respondents from Denmark and Norway tended to protect themselves less than respondents from Sweden, although the perceived seriousness of a tick bite and LB was similar. The effect of perceived seriousness of TBE and country on applying protective measures was less clear.

Women were always more likely than men to wear more protective measures independent of the level of perceived seriousness of a tick bite and LB. However, women were only likely to apply more protective measures than men, when both perceived TBE as ‘very serious’. When the perceived likelihood was the same, females were still more likely to apply protective measures. This corresponds well with what is known about gender and risk perception; women are generally more concerned about hazards than men [46]. Furthermore, females are more easily disgusted by ectoparasites [47] and pathogens [48] resulting in avoidance of the object causing the disgust [49].

The variables ‘perceived likelihood of a tick bite within the next 12 months’ and ‘perceived likelihood of getting LB if bitten by a tick’ on a scale from 0% likelihood to 100% likelihood had a significant effect on applying protective measures. However, the increase in likelihood of protection was very small; for every one unit increase in the perceived likelihood, the probability of using more protection was 0.9% for a tick bite and 1.6% for LB. Nevertheless, it shows a tendency of an increased level of adoption of protective measures based on the perception of probability of an event occurring. Whether or not this variable is generally a good predictor of protection is unclear [27], [31], [50], but based on this study it indicates that informing the public of risk areas and likelihood of getting bitten could potentially lead to a higher level of adoption of protective measures.

Overall, respondents from Denmark and Norway were less likely to apply protective measures than respondents from Sweden regarding all perceived likelihoods. This effect may be explained by different information campaigns regarding ticks or visiting nature, but this was not examined.

However, we will recommend for future research to look into this. An everyday perspective of protection and behaviour might give new answers, as there might be differences in the way people venture into nature. As an example, some areas may be used for different leisure activities in the summer. Naturally wearing long trousers in one setting and wearing open sandals in another setting might be area specific and based on fluctuations during the calendar year. Future research could include qualitative methods such as observation and interviews to uncover the specific practises in order to give recommendations for health promotion and disease prevention.

Finally, we found correlations between how often a respondent is using a protective measure and the perceived efficacy of the same protective measure. For example, a person who believes mosquito repellents do not offer any protection is less likely to use mosquito repellents. Perceived efficacy seems to be a good predictor of protection motivation [19], [28], [51].

Our study shows that the first variable of PMT, here ‘the perceived seriousness of a tick bite’ and to some extent LB, significantly predicts the level of adoption of protective measures applied. The perceived seriousness of TBE, however, did not predict level of adoption of protective measures applied. Although respondents generally have a higher perceived seriousness of TBE than a tick bite and LB [32] the results could be explained by a greater variation in knowledge about TBE because of the patchy distribution of the virus causing the disease. Due to the patchy distribution of TBE-virus (TBEV) in Denmark, Norway, and Sweden, vaccination against TBE is not part of national vaccination programmes in any of the countries. Instead, all three countries recommend the vaccination for people living or working in endemic areas who are at risk of getting tick bites [52–54]. TBEV is much more prevalent in Sweden and Swedish respondents have a better knowledge of TBE compared to respondents in Denmark and Norway [32]. To fully perceive and understand a threat and react on that threat, knowledge and the belief in a benefit from a certain behavior are important [20], yet assessing risk is still difficult [55], and possibly this could explain the results in this study. Another explanation could be vaccinations being available against TBE. If people have a vaccination, even though they perceive TBE as being serious, they may be less likely to apply other protective measures. However, although there are no vaccine registers available for Norway and Sweden, it can be suspected that Sweden has a higher proportion of citizens vaccinated against TBE based on the higher burden there [54] compared to Denmark [52] and Norway [53] which has also recently been shown by self-reporting [56]. It is therefore not supported in this case, that vaccination should lead to a decrease in likelihood of applying other protective measures.

In a revised PMT, self-efficacy is a fourth component that has a positive effect on the adoption of a protective behavior [23]. Unfortunately, this survey did not include a question of self-efficacy of protective measures against LB and TBE and for simplicity the analyses were based on the components of fear appeal in the original protection motivation theory from 1975 [20].

Although this study does not cover self-efficacy, we did examine perceived efficacy of six different protective measures and found a correlation between this and how often a respondent applies the same protective measure. This is in line with another study that found that perceived efficacy is a reliable predictor of preventive measures against ticks [57]. As not all tick-borne pathogens are transmitted equally fast from the tick [58], [59] and some are treatable whereas others are not, the different protective measures offer different levels of protection depending on the pathogen. For example, the risk of contracting LB can be effectively minimized by preventing or shortening tick bites [60]. However, for TBE which is transmitted immediately after the tick bites [59], it is especially clear, why preventing tick bites is important although vaccinations against the virus exist. Knowledge of the existence of this vaccine is different in Denmark, Norway, and Sweden. Fewer than 20% of the respondents from Denmark and Norway knew about the TBE vaccine whereas more than 70% of respondents from Sweden knew about it [32].

As proposed earlier, encouraging the public to take precaution regarding ticks without causing fear that may limit outdoor activities is a challenging task for health authorities [32]. However, results from the present study suggest that a certain level of perceived seriousness and likelihood is required for the motivation of applying protective measures against ticks. Norman et al., 2005 describe how PMT originated in research of the “persuasive impact of fear appeals”. This corresponds well with the original PMT as “the intent to adopt the communicators recommendation is mediated by the amount of protection motivation aroused” [20] which is also in line with other studies [61]. Nevertheless, it is important to distinguish between an irrational fear leading to some people potentially avoiding nature, and a rational fear, or rather, rational cautiousness, still allowing recreational activities in tick areas but while being motivated to protect oneself [62]. An important task in health promotion strategies regarding ticks is therefore to emphasize the actual efficacy of protective measures and benefit of applying different types of protections as well as the actual risk of TBDs [62].

Further, as the cognitive recognition of a fear appeal is dependent on the understanding of a possible fearful event [20] one cannot exclude the importance of education and awareness as emphasized earlier.

A limitation to this study is that respondents answering the survey may have more interest in ticks compared to people who chose not to answer, the use of words subject to personal interpretation such as often and rarely, and the difficulty of assessing a perceived risk or likelihood of something occurring. Further, determining which independent variables are effective in predicting the dependent variable protection is more complex than the few variables examined in this study. The variables ‘perceived seriousness’ and ‘perceived likelihood’ are affected by many other confounding variables such as experience, source of information about ticks and TBDs, occupation, personality, and trust [22], [36], [46]. As well, the survey was lacking questions regarding self-efficacy and the protective measures section was lacking an anti-TBE vaccination option.

Often when people do things with a known high risk of having a negative effect on health (e.g. smoking, overeating) they are often pleasurable [25]; this, however, is not the case in protecting oneself against ticks. But looking silly, forgetfulness, indifference, inconvenience compared to the risk are possible reasons why people don’t apply protective measures. Examining these variables could lead to a better understanding of people’s behavior regarding protection against ticks. Future surveys examining public knowledge and protective behavior regarding ticks should include self-efficacy. This may be a variable that better predicts protection against ticks [23] as it has been found to be the case in other studies and a change in peoples’ perceptions of self-efficacy may be an important part of health promotion strategies [36].???????

## Conclusions

Our results indicate that variables of Protection Motivation Theory may be useful in predicting the level of adoption of protection applied against ticks in Denmark, Norway, and Sweden and possibly other countries where tick-borne diseases are of concern.

Further, our results confirm that women are more likely to be protecting themselves than men, even when the perceived level of seriousness and likelihood of a tick bite and LB are the same in the two groups. Perceived seriousness and likelihood of TBE were not good predictors. This could be caused by TBE being rarer in occurrence and people having poor knowledge about the disease. People from Sweden were more likely to protect themselves than people from Denmark and Norway when TBE was considered very serious and when considering the perceived likelihood of getting TBE if bitten by a tick.

Being able to clearly communicate the seriousness of tick and TBDs, likelihood of a TBD, combined with the efficacy of protective measures to the public, is important in tick prevention strategies.

## Electronic supplementary material

Below is the link to the electronic supplementary material.

Supplementary Material 1

## Data Availability

The questions from the survey used in this study are attached as supplementary information. The datasets used and/or analysed during the current study are available from Karen A. Krogfelt (karenak@ruc.dk) on reasonable request.

## Abbreviations

LB: Lyme Borreliosis
TBE: Tick-Borne Encephalitis
TBD: Tick-Borne Disease
PMT: Protection Motivation Theory

## References

[1] WHO., “Vector-borne diseases,” Fact sheets, Mar. 02, 2020. https://www.who.int/news-room/fact-sheets/detail/vector-borne-diseases (accessed Jan. 12, 2022).

[2] European Centre for Disease Prevention and Control (ECDC)., “European centre for Disease Prevention and Control (ECDC).” https://www.ecdc.europa.eu/en/climate-change/climate-change-europe/vector-borne-diseases (accessed Mar 26, 2023).

[3] Center for Disease Control (CDC)., “Center for Disease Control (CDC).” https://www.cdc.gov/ncezid/dvbd/index.html (accessed Mar. 26, 2023).

[4] Sykes RA, Makiello P. “An estimate of Lyme borreliosis incidence in Western Europe,” J. Public Health (Bangkok)., vol. 39, no. 1, pp. 74–81, Mar. 2017, 10.1093/PUBMED/FDW017. 10.1093/pubmed/fdw017 26966194

[5] Schwartz AM, Kugeler KJ, Nelson CA, Marx GE, Hinckley AF (2021).

[6] Gynthersen RMM, et al. Classification of patients referred under suspicion of tick-borne diseases, Copenhagen, Denmark. Ticks Tick Borne Dis. Jan. 2021;12(1):101591. 10.1016/J.TTBDIS.2020.101591. 10.1016/j.ttbdis.2020.101591 33126203

[7] Rebman AW, Yang T, Yoon I, Powell D, Geller SA, Aucott JN. “Initial Presentation and Time to Treatment in Early Lyme Disease,” Am. J. Trop. Med. Hyg., vol. 1, no. aop, Feb. 2023, 10.4269/AJTMH.22-0437. 10.4269/ajtmh.22-0437 PMC10077021 36746657

[8] Knudtzen FC, Andersen NS, Jensen TG, Skarphédinsson S. “Characteristics and Clinical Outcome of Lyme Neuroborreliosis in a High Endemic Area, 1995–2014: A Retrospective Cohort Study in Denmark,” Clin. Infect. Dis., vol. 65, no. 9, pp. 1489–1495, Oct. 2017, 10.1093/CID/CIX568. 10.1093/cid/cix568 29048514

[9] Hirsch AG, et al. Risk factors and outcomes of treatment delays in Lyme Disease: a Population-Based Retrospective Cohort Study. Front Med. Nov. 2020;7:560018. 10.3389/FMED.2020.560018/FULL. 10.3389/fmed.2020.560018 PMC7726265 33324657

[10] Henningsson AJ, Malmvall BE, Ernerudh J, Matussek A, Forsberg P (2010). Neuroborreliosis–an epidemiological, clinical and healthcare cost study from an endemic area in the south-east of Sweden. Clin Microbiol Infect.

[11] Connally NP, Durante AJ, Yousey-Hindes KM, Meek JI, Nelson RS, Heimer R. “Peridomestic Lyme Disease Prevention. Results of a Population-Based Case-Control Study,” Am. J. Prev. Med., vol. 37, no. 3, pp. 201–206, Sep. 2009, 10.1016/j.amepre.2009.04.026. 10.1016/j.amepre.2009.04.026 19595558

[12] des Vignes F, Piesman J, Heffernan R, Schulze TL, Stafford KC, Fish D. “Effect of tick removal on transmission of Borrelia burgdorferi and Ehrlichia phagocytophila by Ixodes scapularis Nymphs,” J. Infect. Dis., vol. 183, no. 5, pp. 773–778, Mar. 2001, 10.1086/318818. 10.1086/318818 11181154

[13] Kahl O, Janetzki-Mittmann C, Gray JS, Jonas R, Stein J, De Boer R (1998). Risk of infection with Borrelia burgdorferi sensu lato for a host in relation to the duration of nymphal Ixodes ricinus feeding and the method of tick removal. Zentralblatt fur Bakteriol.

[14] Schwartz AM, Mackeprang JM, Mead PS, Hinckley AF. “Effectiveness of personal protection measures against Lyme disease: A review of epidemiologic studies from the United States,” Zoonoses Public Health, vol. 69, no. 7, pp. 777–791, Nov. 2022, 10.1111/ZPH.12984. 10.1111/zph.12984 35791092

[15] Jepsen MT, Jokelainen P, Jore S, Boman A, Slunge D, Krogfelt KA. Protective practices against tick bites in Denmark, Norway and Sweden: a questionnaire-based study. BMC Public Health. 2019;19(1). 10.1186/s12889-019-7613-4. 10.1186/s12889-019-7613-4 PMC6805683 31640665

[16] Zöldi V, Turunen T, Lyytikäinen O, Sane J. Knowledge, attitudes, and practices regarding ticks and tick-borne diseases, Finland. Ticks Tick Borne Dis. Oct. 2017;8:872–7. 10.1016/J.TTBDIS.2017.07.004. 10.1016/j.ttbdis.2017.07.004 28778675

[17] Buczek A, Pilch J, Buczek W. “Tick preventive behaviors and practices adopted by medical students from Poland, Germany, and Thailand in relation to socio-demographic conditions and their knowledge of ticks and tick-borne diseases,” Insects, vol. 11, no. 12, pp. 1–17, Dec. 2020, 10.3390/INSECTS11120863. 10.3390/insects11120863 PMC7761883 33287425

[18] Valente SL, Wemple D, Ramos S, Cashman SB, Savageau JA. “Preventive behaviors and knowledge of tick-borne illnesses: results of a survey from an endemic area,” J Public Heal Manag Pract, vol. 21, no. 3, pp. E16–E23, Dec. 2015. 10.1097/PHH.0000000000000098 24762630

[19] Beaujean DJMA, Bults M, van Steenbergen JE, Voeten HACM. “Study on public perceptions and protective behaviors regarding Lyme disease among the general public in the Netherlands: implications for prevention programs,” BMC Public Heal. 2013 131, vol. 13, no. 1, pp. 1–11, Mar. 2013, 10.1186/1471-2458-13-225. 10.1186/1471-2458-13-225 PMC3602656 23497311

[20] Rogers RW (1975). A Protection Motivation Theory of Fear Appeals and attitude change. J Psychol no.

[21] Adolphs R. The Biology of Fear. Curr Biol. Jan. 2013;23(2):R. 10.1016/J.CUB.2012.11.055. 10.1016/j.cub.2012.11.055 PMC3595162 23347946

[22] Rogers RW. “Cognitive and physiological processes in fear appeals and attitude change: a revised theory of protection motivation,” in Basic Social Psychophysiological Research, 1983, pp. 153–76.

[23] Maddux JE, Rogers RW. “Protection Motivation and Self-Efficacy: A Revised Theory of Fear Appeals and Attitude Change,” 1983.

[24] Norman P, Boer H, Seydel ER, Mullan B. In: Conner M, Norman P, editors. Protection Motivation Theory,” in Predicting Health Behaviour. 2nd ed. Open University Press; 2005. pp. 81–126.

[25] Ferrer RA, Klein WMP. Risk perceptions and health behavior. Curr Opin Psychol. Oct. 2015;5:85–9. 10.1016/J.COPSYC.2015.03.012. 10.1016/j.copsyc.2015.03.012 PMC4525709 26258160

[26] Rogers RW, Prentice-Dunn S. Protection Motivation Theory. In: Gochman DS, editor. in Handbook of health behavior research 1: personal and social determinants. Plenum Press; 1997. pp. 113–32.

[27] Parisi S, Mazigo HD, Kreibich S, Puchner K, Kasang C, Mueller A. Factors associated with relevant knowledge of intestinal schistosomiasis and intention to participate in treatment campaigns: a cross sectional survey among school children at Ijinga Island on Lake Victoria, North-Western Tanzania. BMC Public Health. Dec. 2019;19(1). 10.1186/S12889-019-8091-4. 10.1186/s12889-019-8091-4 PMC6937638 31888548

[28] Anderson KR, Naaman K, Omodior E, Karikari G, Pennington-Gray L, Omodior O (2020). Predicting Chikungunya disease personal protective behaviors: results of a cross-sectional survey of US-Caribbean travelers. Heal Promot Perspect.

[29] Xiao H, et al. Protection motivation theory in predicting intention to engage in protective behaviors against schistosomiasis among middle school students in rural China. PLoS Negl Trop Dis. 2014;8(10). 10.1371/JOURNAL.PNTD.0003246. 10.1371/journal.pntd.0003246 PMC4199519 25329829

[30] Floyd DL, Prentice-Dunn S, Rogers RW. “A Meta-Analysis of Research on Protection Motivation Theory,” 2000.

[31] Milne S, Sheeran P, Orbell S (2000). Prediction and intervention in health-related behavior: a meta-analytic review of protection motivation theory. J Appl Soc Psychol.

[32] Slunge D, Jore S, Krogfelt KA, Jepsen MT, Boman A. Who is afraid of ticks and tick-borne diseases? Results from a cross-sectional survey in Scandinavia. BMC Public Health. Dec. 2019;19(1). 10.1186/s12889-019-7977-5. 10.1186/s12889-019-7977-5 PMC6907266 31829150

[33] Niesobecki S, et al. Knowledge, attitudes, and behaviors regarding tick-borne disease prevention in endemic areas. Ticks Tick Borne Dis. Oct. 2019;10(6):101264. 10.1016/J.TTBDIS.2019.07.008. 10.1016/j.ttbdis.2019.07.008 PMC10948045 31431351

[34] Mowbray F, AmlÔt R, Rubin GJ. “Predictors of protective behaviour against ticks in the UK: A mixed methods study,” Ticks Tick. Borne. Dis., vol. 5, no. 4, pp. 392–400, Jun. 2014, 10.1016/J.TTBDIS.2014.01.006. 10.1016/j.ttbdis.2014.01.006 24713278

[35] Slunge D, Boman A. Learning to live with ticks? The role of exposure and risk perceptions in protective behaviour against tick-borne diseases. PLoS ONE. Jun. 2018;13(6). 10.1371/journal.pone.0198286. 10.1371/journal.pone.0198286 PMC6010238 29924806

[36] Norman P, Boer H, Seydel ER, Mullan B. “Protection Motivation Theory,” in Predicting and changing Health Behaviour: Research and practice with Social Cognition Models, 3rd ed., McGraw-Hill Education, 2015, 70–106.

[37] Eisen RJ, Piesman J, Zielinski-Gutierrez E, Eisen L. “What Do We Need to Know About Disease Ecology to Prevent Lyme Disease in the Northeastern United States?,” J. Med. Entomol., vol. 49, no. 1, pp. 11–22, Jan. 2012, 10.1603/ME11138. 10.1603/me11138 22308766

[38] Piesman J, Eisen L (2008). Prevention of tick-borne diseases. Annu Rev Entomol.

[39] Poland GA. “Prevention of Lyme Disease: A Review of the Evidence,” Mayo Clin. Proc., vol. 76, no. 7, pp. 713–724, Jul. 2001, 10.4065/76.7.713. 10.4065/76.7.713 11444404

[40] Jore S, et al. Spatial tick bite exposure and associated risk factors in Scandinavia. Infect Ecol Epidemiol. 2020;10(1). 10.1080/20008686.2020.1764693. 10.1080/20008686.2020.1764693 PMC7448850 32922687

[41] Andreassen A (2012). Prevalence of tick borne encephalitis virus in tick nymphs in relation to climatic factors on the southern coast of Norway. Parasit Vectors.

[42] Andersen NS, et al. Phylogenetic characterization of tick-borne encephalitis virus from Bornholm, Denmark. Ticks Tick Borne Dis. Apr. 2019;10(3):533–9. 10.1016/j.ttbdis.2018.12.008. 10.1016/j.ttbdis.2018.12.008 30704909

[43] Fomsgaard A et al. “Tick-borne Encephalitis Virus, Zealand, Denmark, 2011,” Emerg. Infect. Dis., vol. 19, no. 7, p. 1171, Jul. 2013, 10.3201/EID1907.130092. 10.3201/eid1907.130092 PMC3903456 23764123

[44] Pettersson JHO, Golovljova I, Vene S, Jaenson TGT. “Prevalence of tick-borne encephalitis virus in Ixodes ricinus ticks in northern Europe with particular reference to Southern Sweden,” Parasit. Vectors, vol. 7, no. 1, p. 102, Mar. 2014, 10.1186/1756-3305-7-102. 10.1186/1756-3305-7-102 PMC4007564 24618209

[45] Kjær LJ et al. “Spatial patterns of pathogen prevalence in questing Ixodes ricinus nymphs in southern Scandinavia, 2016,” Sci. Rep., vol. 10, no. 1, Dec. 2020, 10.1038/s41598-020-76334-5. 10.1038/s41598-020-76334-5 PMC7652892 33168841

[46] Slovic P. Informing and educating the public about risk. In: Löfstedt RE, editor. in The perception of risk. Earthscan Publications Ltd; 2000. pp. 182–98.

[47] Prokop P, Fančovičová J (2010). The association between disgust, danger and fear of macroparasites and human behaviour. Acta Ethol.

[48] Al-Shawaf L, Lewis DMG, Buss DM. “Sex Differences in Disgust: Why Are Women More Easily Disgusted Than Men?,” Emot. Rev., vol. 10, no. 2, pp. 149–160, Apr. 2018, 10.1177/1754073917709940/FORMAT/EPUB.

[49] Rozin P, Haidt J, McCauley CR, Lewis M, Haviland-Jones JM (2000). Disgust. Handbook of emotions.

[50] Downing ST, Mccarty RJ, Guastello AD, Cooke DL, Mcnamara JPH (2022). Assessing the predictors of adaptive and maladaptive Covid-19 preventive behaviours: an application of protection motivation theory. Psychol Heal Med.

[51] Butler AD, Sedghi T, Petrini JR, Ahmadi R. Tick-borne disease preventive practices and perceptions in an endemic area. Ticks Tick Borne Dis. Mar. 2016;7(2):331–7. 10.1016/j.ttbdis.2015.12.003. 10.1016/j.ttbdis.2015.12.003 26704290

[52] Danish Public Health Agency., “Danish Public Health Agency.” https://www.ssi.dk/sygdomme-beredskab-og-forskning/sygdomsleksikon/t/tbe (accessed Mar. 26, 2023).

[53] Norwegian Public Health Agency., “Norwegian Public Health Agency.” https://www.fhi.no/nettpub/vaksinasjonsveilederen-for-helsepersonell/vaksiner-mot-de-enkelte-sykdommene/skogflattencefalittvaksinasjon-tbe-/ (accessed Mar. 26, 2023).

[54] Swedish Public Health Agency., “Swedish Public Health Agency.” https://www.folkhalsomyndigheten.se/smittskydd-beredskap/smittsamma-sjukdomar/tick-borne-encephalitis-tbe/ (accessed Mar. 26, 2023).

[55] Slovic P, Fischhoff B, Lichtenstain S. Rating the risks. In: Löfstedt RE, editor. The perception of risk. earthscan Publications Ltd; 2000. pp. 104–20.

[56] Pilz A, Erber W, Schmitt HJ. Vaccine uptake in 20 countries in Europe 2020: focus on tick-borne encephalitis (TBE). Ticks Tick Borne Dis. Jan. 2023;14(1):102059. 10.1016/J.TTBDIS.2022.102059. 10.1016/j.ttbdis.2022.102059 36410164

[57] Aenishaenslin C, et al. Factors associated with preventive behaviors regarding Lyme disease in Canada and Switzerland: a comparative study. BMC Public Health. Dec. 2015;15(1). 10.1186/s12889-015-1539-2. 10.1186/s12889-015-1539-2 PMC4349712 25884424

[58] Cook MJ. Lyme borreliosis: a review of data on transmission time after tick attachment. Int J Gen Med. Dec. 2015;8:1. 10.2147/IJGM.S73791. 10.2147/IJGM.S73791 PMC4278789 25565881

[59] Alekseev A, Chunikhin SP. “The experimental transmission of the tick-borne encephalitis virus by ixodid ticks (the mechanisms, time periods, species and sex differences),” Parazitologiia, vol. 24, no. 3, pp. 177–185, May 1990, Accessed: Sep. 02, 2021. [Online]. Available: https://pubmed.ncbi.nlm.nih.gov/2216530/. 2216530

[60] Cook MJ (2015). Lyme-borreliosis: a review of data on transmission time after tick attachment. Int J Gen Med.

[61] Sheeran P, Harris PR, Epton T. “Does heightening risk appraisals change people’s intentions and behavior? A meta-analysis of experimental studies,” Psychol. Bull., vol. 140, no. 2, pp. 511–543, Mar. 2014, 10.1037/a0033065. 10.1037/a0033065 23731175

[62] Kok G, Bartholomew LK, Parcel GS, Gottlieb NH, Fernández ME. Finding theory- and evidence-based alternatives to fear appeals: intervention mapping. Int J Psychol. Apr. 2014;49(2):98. 10.1002/IJOP.12001. 10.1002/ijop.12001 PMC4255304 24811880

