# Supplementary material for: Can protection motivation theory predict protective behavior against ticks?
Source: BMC Public Health. 2023 Jun 22;23:1214. doi: 10.1186/s12889-023-16125-5 (PMC10286392; doi:10.1186/s12889-023-16125-5)
Supplement: Supplementary file 1 — Supplementary Material 1 [file 12889_2023_16125_MOESM1_ESM.docx]

Appendix 1

**Survey on risk perceptions, knowledge, and behaviour related to ticks and tick-borne diseases**

*[This is an English translation of parts of the questionnaire in Swedish which was sent out to respondents in Sweden in October 2016 as part of the ScandTick Innovation project. Similar surveys were sent out simultaneously in Danish and Norwegian to respondents in Denmark and Norway. Please note that this was a web-based questionnaire. Routing was used so that all respondents were not presented with all the questions. The questionnaire was translated by a professional language editor contracted by the University of Gothenburg. We have only disclosed the questions that were used for this study.]*

[qGender – CATEGORICAL – single – Must answer]
Gender:

- (_1) Woman
- (_2) Man
- (_3) Other
- (_4) I choose not to respond

[q20 – GRID – single – Must answer]
To what extent do you take any of the following actions <i>to protect yourself against tick bites</i> when spending time in the outdoors or in other places where you may be exposed to ticks? <i>Please give one response per suggested action.</i> {#ex_script}

|  | Never | Rarely | Often | Always |
| --- | --- | --- | --- | --- |
| I wear clothes that cover my entire legs and arms | ⃝ | ⃝ | ⃝ | ⃝ |
| I use mosquito or tick repellants | ⃝ | ⃝ | ⃝ | ⃝ |
| I tuck my trousers into my socks | ⃝ | ⃝ | ⃝ | ⃝ |
| I avoid tall grass and walking near bushes | ⃝ | ⃝ | ⃝ | ⃝ |
| I check my body and clothes for ticks when visiting areas where I may be exposed to them | ⃝ | ⃝ | ⃝ | ⃝ |
| I check my body for ticks after visiting areas where I may have been exposed to them | ⃝ | ⃝ | ⃝ | ⃝ |
| I check my pet(s) for ticks | ⃝ | ⃝ | ⃝ | ⃝ |

[q22 – GRID – single – Must answer]
What level of protection against tick bites and tick-borne diseases do you believe the following actions provide? <i>Please provide one response per action.</i>{#ex_script}

|  | No protection | Weak protection | Fairly strong protection | Very strong protection |
| --- | --- | --- | --- | --- |
| Wearing clothes that cover one’s entire legs and arms | ⃝ | ⃝ | ⃝ | ⃝ |
| Using mosquito or tick repellants | ⃝ | ⃝ | ⃝ | ⃝ |
| Tucking trousers into socks | ⃝ | ⃝ | ⃝ | ⃝ |
| Avoiding tall grass and not walking near bushes | ⃝ | ⃝ | ⃝ | ⃝ |
| Checking one’s body and clothes for ticks when being outside | ⃝ | ⃝ | ⃝ | ⃝ |
| Checking one’s body for ticks after having been outside | ⃝ | ⃝ | ⃝ | ⃝ |
| Checking pets for ticks | ⃝ | ⃝ | ⃝ | ⃝ |

[q23A – GRID – single – Must answer]
How serious do you believe it is to get bit by a tick? <i> Please respond on a scale from 0 to 10, where 0 = "not serious at all” and 10 = “very serious”.</i>

|  | 0 = Not serious at all | 1 | 2 | 3 | 4 | 5 | 6 | 7 | 8 | 9 | 10 = Very serious | Don't know |
| --- | --- | --- | --- | --- | --- | --- | --- | --- | --- | --- | --- | --- |
|  | ⃝ | ⃝ | ⃝ | ⃝ | ⃝ | ⃝ | ⃝ | ⃝ | ⃝ | ⃝ | ⃝ | ⃝ |

[q23B – GRID – single – Must answer]
How serious do you believe it is to get the tick-borne disease called Lyme borreliosis? <i> Please respond on a scale from 0 to 10, where 0 = "not serious at all” and 10 = “very serious”.</i>

|  | 0 = Not serious at all | 1 | 2 | 3 | 4 | 5 | 6 | 7 | 8 | 9 | 10 = Very serious | Don't know |
| --- | --- | --- | --- | --- | --- | --- | --- | --- | --- | --- | --- | --- |
|  | ⃝ | ⃝ | ⃝ | ⃝ | ⃝ | ⃝ | ⃝ | ⃝ | ⃝ | ⃝ | ⃝ | ⃝ |

[q23C – GRID – single – Must answer]
How serious do you believe it is to get the tick-borne disease called TBE (tick-borne encephalitis)? <i> Please respond on a scale from 0 to 10, where 0 = "not serious at all” and 10 = “very serious”.</i>

|  | 0 = Not serious at all | 1 | 2 | 3 | 4 | 5 | 6 | 7 | 8 | 9 | 10 = Very serious | Don't know |
| --- | --- | --- | --- | --- | --- | --- | --- | --- | --- | --- | --- | --- |
|  | ⃝ | ⃝ | ⃝ | ⃝ | ⃝ | ⃝ | ⃝ | ⃝ | ⃝ | ⃝ | ⃝ | ⃝ |

[q24 – INFO – single – Optional]
How likely are you to get bit by a tick in the next 12 months? <i>Please estimate the likelihood in per cent from 0 to 100 and write the value in the field below, where 0 = “I’m absolutely certain I will not get bit” and 100 = “I’m absolutely certain I will get bit”.</i>

- (NotMust)

[q25 – INFO – single – Optional]
If you were to get bitten by a tick, how likely do you think you would be to get Lyme borreliosis? <i> Please estimate the likelihood in per cent from 0 to 100 and write the value in the field below, where 0 = “I’m absolutely certain I would not get Lyme borreliosis” and 100 = “I’m absolutely certain I would get Lyme borreliosis”.</i>

- (NotMust)

[q26 – INFO – single – Optional]
If you were to get bitten by a tick, how likely do you think you would be to get TBE (tick-borne encephalitis)? <i> Please estimate the likelihood in per cent from 0 to 100 and write the value in the field below, where 0 = “I’m absolutely certain I would not get TBE” and 100 = “I’m absolutely certain I would get TBE”'</i>

- (NotMust)
